# Heterochromatin distribution and comparative karyo-morphological studies in *Vigna umbellata* Thunberg, 1969 and *V. aconitifolia* Jacquin, 1969 (Fabaceae) accessions

**DOI:** 10.3897/CompCytogen.v9i1.9012

**Published:** 2015-03-31

**Authors:** Anju Shamurailatpam, Latha Madhavan, Shrirang Ramachandra Yadav, Kangila Venkatraman Bhat, Satyawada Rama Rao

**Affiliations:** 1 Department of Biotechnology and Bioinformatics, North Eastern Hill University, Shillong-793022, India; 2 National Bureau of Plant Genetic Resources, Regional Station, Vellanikkara, Thrissur-680654, India; 3 Department of Botany, Shivaji University, Kolhapur-416004, India; 4 National Research Centre on DNA Fingerprinting, National Bureau of Plant Genetic Resources, Pusa, New Delhi-110012, India

**Keywords:** Asymmetry index, C-heterochromatin, Fabaceae, karyotype, NOR-chromosomes, *Vigna*

## Abstract

Chromosome studies along with heterochromatin distribution pattern analysis have been carried out in two domesticated species of *Vigna* Savi, 1824 which grow in contrasting geo-climatic conditions of India: *Vigna umbellata* Thunberg, 1969, a legume well acclimatized to subtropical hilly regions of North-east India and *Vigna aconitifolia* Jacquin, 1969, a species of arid and semi-arid regions in desert plains of Western India. Karyo-morphological studies in both species reveal 2*n* = 22 chromosomes without any evidence of numerical variation and the overall karyotype symmetry in chromosome morphology suggest that the diversification at intraspecific level in genus *Vigna* has occurred through structural alteration of chromosomes, rather than numerical changes. Heterochromatin distribution as revealed by fluorochrome binding pattern using CMA_3_ and DAPI, confirms the occurrence of relatively more GC content in *Vigna aconitifolia* as compared to *Vigna umbellata*. However, AT content was found to be comparatively higher in *Vigna umbellata* which perhaps play a role in species interrelationships.

## Introduction

The pantropical genus *Vigna* Savi, 1824 (Fabaceae) includes 104 described species (Lewis et al. 2005). Among its subgenera, only *Ceratotropis* Marechal, 1978 is known for its rich species diversity in Asia (Verdcourt 1970, Marechal et al. 1978, Tateishi 1996). Tomooka et al. (2002) recognized 21 species in the subgenus *Ceratotropis*, out of which six species are domesticated: azuki bean (*Vigna angularis* Willdenow, 1969), mung bean (*Vigna radiata* Linnaeus, 1954), black gram (*Vigna mungo* Linnaeus, 1956), rice bean (*Vigna umbellata* Thunberg, 1969), moth bean (*Vigna aconitifolia* Jacquin, 1969) and creole bean (Vigna reflexo-pilosa var. glabra Marechal, 1911). The genetic resources and diversity in cultivated and wild forms of subgenus *Ceratotropis* occurring in Indian subcontinent are extremely rich and interesting (Bisht et al. 2005). The domesticated *Vigna aconitifolia* is confined only to the tropical region of India, while *Vigna umbellata* is widely domesticated across the South-east Asia. The origin of *Vigna umbellata* is considered to be Indo-China region and also to a certain extent from South-east Asia (Marechal et al. 1978, Baudoin and Marechal 1988).

The structure and morphology of the chromosomes are of vital importance when studying the origin, evolution and classification of taxa (Yang et al. 2005) as well as distance or relatedness among diverse genomes (Stace 2000, Kumar and Rao 2002). Quite a few number of reports dealing with such studies are available for *Vigna* species (Rao and Chandel 1991, Rao and Raina 2004, Shamurailatpam et al. 2012).

Chromosome location and characterization of C-heterochromatin by fluorescence staining procedures which preferentially stain GC-rich DNA and DAPI, which localised AT-rich regions has been successfully applied in a large number of Fabaceae taxa including *Cicer arietinum* Linnaeus, 1753 (Galasso et al. 1996a); *Phaseolus calcaratus* Roxburgh, 1832 (Zheng et al. 1991); *Sesbania tetraptera* Hochstetter, 1871 (Forni-Martins et al. 1994, Forni-Martins and Guerra 1999); *Vicia faba* Linnaeus, 1753 (Greilhuber 1975); *Vigna ambacensis* Welwitsch, 1978 (Galasso et al. 1996b).

A certain degree of chromosomal variation at inter-specific level of the genus *Vigna* has been documented using cytogenetic approaches by earlier workers (Rao and Chandel 1991, Shamurailatpam et al. 2012). Hence, it will be quite significant to see the extent of variation among the domesticated species of Vigna (Ceratotropis). *Vigna umbellata* is a species domesticated extensively in the subtropical hilly and moist regions of North-east India. On the other hand, *Vigna aconitifolia* has been adapted to the arid and semi-arid region of tropical Western plain of India. Analysis of karyo-morphological details in *Vigna umbellata* and *Vigna aconitifolia*, adapted to extremely contrasting environmental conditions, may ultimately help us to define their chromosome variation. Meaningful propagation programs can be developed from such information.

## Materials and methods

Karyo-morphological studies were undertaken in ten accessions each of *Vigna umbellata* and *Vigna aconitifolia*. The germplasm has been obtained from Indian Council of Agricultural Research (ICAR), Baranapi, Meghalaya and also from National Bureau of Plant Genetic Resources (NBPGR), New Delhi. Actively growing root tips of about 1–2 cm long were excised from germinating seeds on moist filter paper in Petri dishes at 25 ± 2 °C, pre-treated with 0.025% colchicine (Himedia) for 3 h at room temperature (20 ± 2 °C). The root tips after pre-treatment were fixed in freshly prepared ethanol-acetic acid (v/v, 3:1) and subsequently stored at 4 °C until required. For slide preparation, the root tips were washed twice in distilled water, hydrolyzed in 1N HCl at 60 °C for 8 min and stained in Feulgen stain (leuco-basic fuchsin) for 45 min. The stained root tips were thoroughly washed and subsequently squashed in 1% acetocarmine. The micro-photographs of the metaphase plates were taken from both temporary and permanent preparations. At least 10–15 clear preparations of chromosome complements of each species were analyzed. Photo-idiograms were prepared from photomicrographs by cutting out individual chromosome and arranging them in descending order of their length and matching on the basis of morphology, the chromosomes were resolved into 11pairs. The standard method of chromosome classification given by Battaglia (1955) classification of metacentric / median (V), submetacentric/ submedian (L), subtelocentric (J) and telocentric (I) based on the arm ratio of 1:1, >1:1<1.3, >1:3<1:0 and 1:0 respectively was employed for comparison. The degree of asymmetry was estimated by means of the parameters proposed by Peruzzi and Eroğlu (2013): Coefficient of Variation of Chromosome Length (CV_CL_) and Mean Centromeric Asymmetry (M_CA_).

For heterochromatin characterization, root-tips were digested in 2% cellulase and 20% pectinase solution for 180 min at 37°C. Meristems were washed in distilled water, squashed in a drop of 45% acetic acid, and frozen in liquid nitrogen. The slides were stained with DAPI (2 µg/ml): glycerol (1:1, v/v) solution to allow selection of the best plates. Subsequently, they were destained in ethanol: glacial acetic acid (3:1, v/v) for 30 min and transferred to absolute ethanol for 1 h, both at room temperature. Slides were air-dried and aged for 3 days at room temperature. The slides were stained with CMA_3_ (0.5 mg/ml, 1 h) and DAPI (2 µg/ml, 30 min), mounted in McIlvaine’s buffer (pH 7.0): glycerol (1:1, v/v), and stored for 3 days (Schweizer and Ambros 1994). Slides were analyzed under Leica DM 4000 B microscope and photographs were carried out with different filter combinations using Leica CCD camera.

## Results

**Figure 1.**
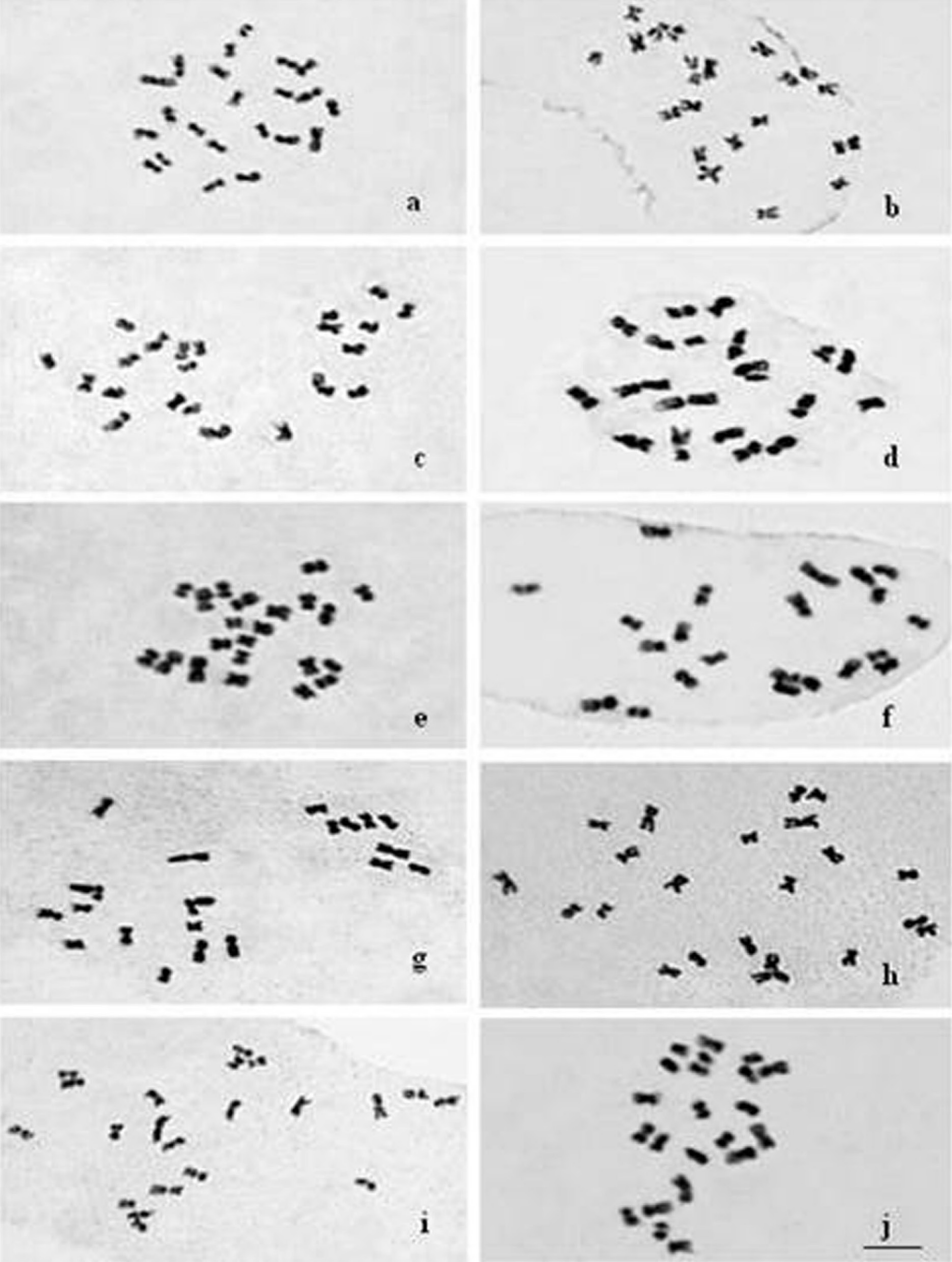

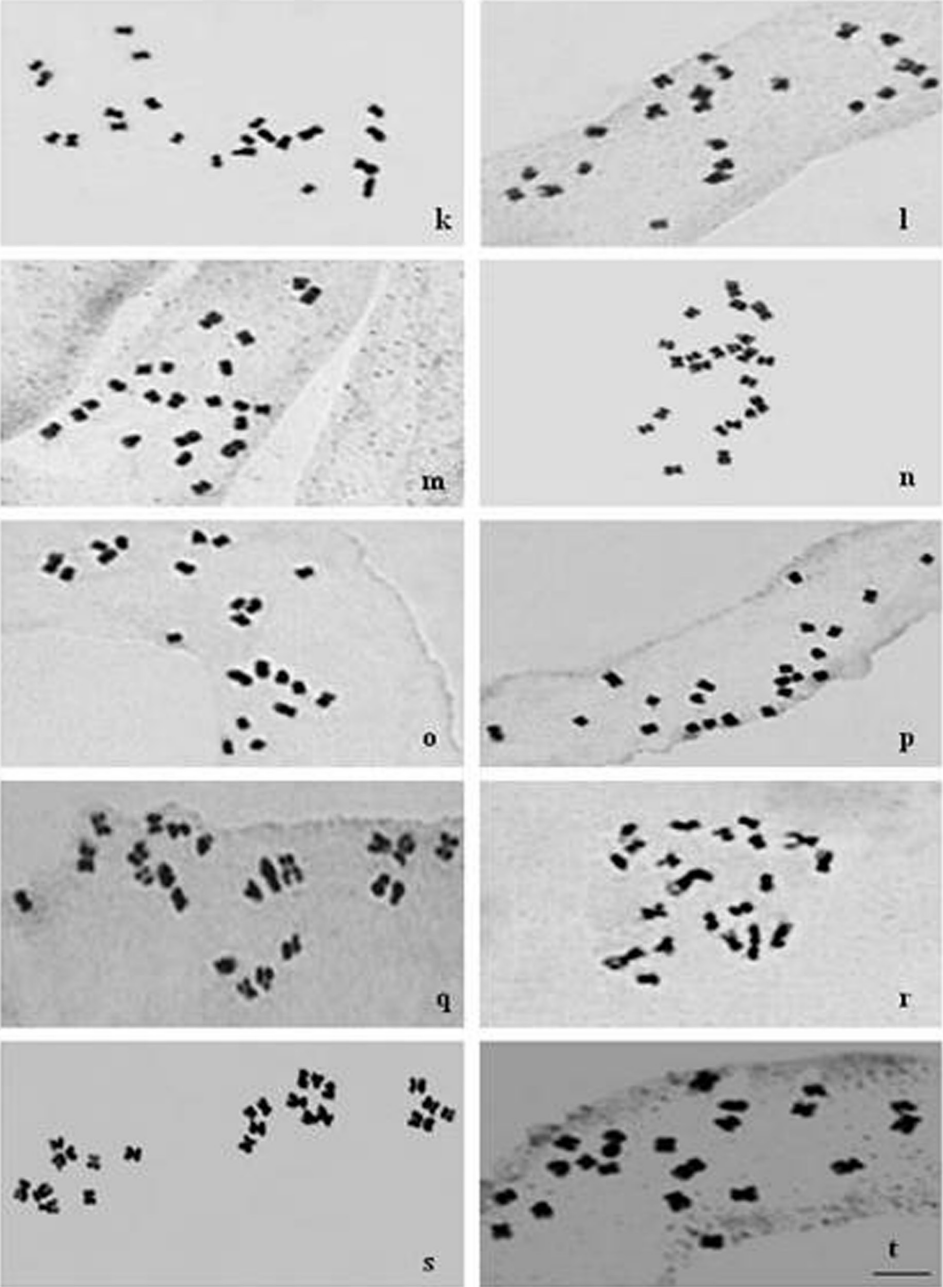
Mitotic complements of 10 accessions of *Vigna umbellata*. **a–j:** **a** BKSB 205 **b** TRB 160 **c** RBS 35 **d** IC 551699 **e** BKSB 192 **f** RBS 53 **g** IC 55440 **h** IC 176563 **i** EC 97882 **j** BKSB 194 **k–t:** **k** IC 36157 **l** VDV 6175 **m** IC 472147 **n** RM 040 **o** IC 39809 **p** IC 285159 **q** IC 36592 **r** IC 472173 s IC 39713 **t** IC 36562. Bar = 5 μm.

The somatic chromosome number of all the accessions had consistently 2*n* = 2*x* = 22 (Fig. 1). The chromosome complements were resolved into 11 pairs which formed a graded series from longest to shortest within the idiograms. A noticeable difference in length between the longest and the shortest chromosomes within the complement was recorded (Table 1). The longest chromosome of the haploid complement was almost 2.5 times longer than the shortest one in *Vigna aconitifolia* accessions, while it was 2 times longer than the shortest one in *Vigna umbellata* accessions. Further investigated accessions belonging to *Vigna umbellata* and *Vigna aconitifolia* had metacentric, submetacentric and subtelocentric chromosomes in their respective chromosome complements. Submetacentric chromosomes outnumbered the metacentric ones in *Vigna aconitifolia* accessions while metacentric chromosomes outnumbered the submetacentric chromosomes in the case of *Vigna umbellata* accessions.

**Figure 2.**
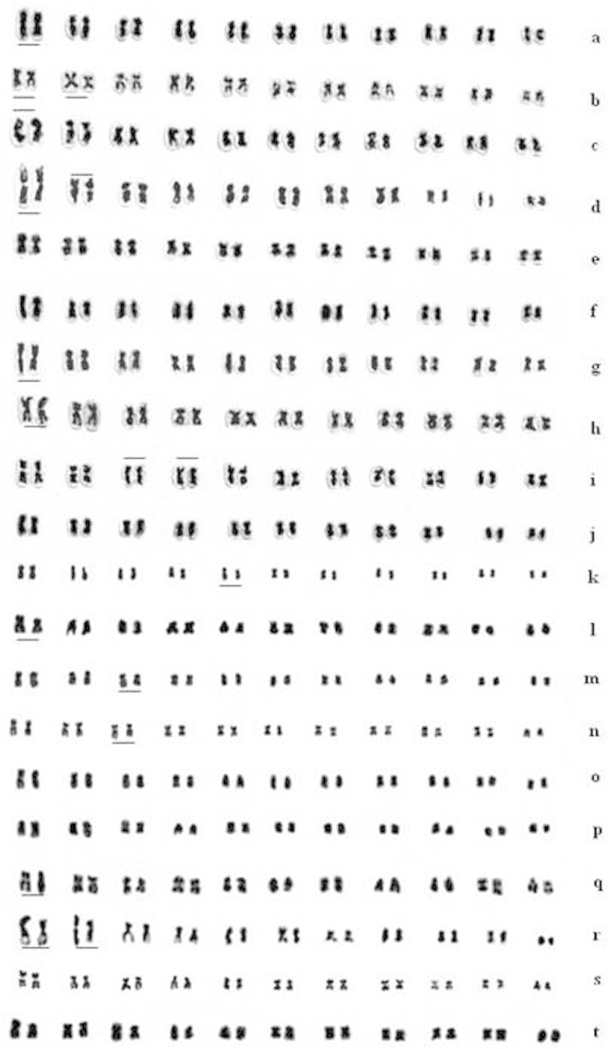
Photo-idiograms of **a–j** 10 accessions of *Vigna umbellata* **a** BKSB 205 **b** TRB 160 **c** RBS 35 **d** IC 551699 **e** BKSB 192, **f** RBS 53 **g** IC 55440 **h** IC 176563 **i** EC 97882 **j** BKSB 194 **k–t** 10 accessions of *Vigna aconitifolia* **k** IC 36157 **l** VDV 6175 **m** IC 472147 **n** RM 040 **o** IC 39809 **p** IC 285159 **q** IC 36592 **r** IC 472173 **s** IC 39713 **t** IC 36562. Heteromorphic groups marked above the short arm and nucleolar groups are marked below the long arm.

Various accessions of these species have shown distinctive variation in the karyotype with respect to number of metacentric and submetacentric chromosomes (Fig. 2). Subtelocentric chromosomes were found in *Vigna aconitifolia* but not in *Vigna umbellata* accessions. Heteromorphic chromosome and nucleolar chromosomes are recorded in the accessions of both *Vigna umbellata* and *Vigna aconitifolia*.

**Table 1.** Karyomorphology and arm ratio in studied taxa of *Vigna*.

Sl. no.	Species	Accessions no.	2n	Chromosome arm length (L/S ratio)											Ratio of longest and shortest chromosome	Karyotype formula
				I	II	III	IV	V	VI	VII	VIII	IX	X	XI		
1	*Vigna umbellata*	BKSB 205	22	1.12 1.14	1.8	1.5	1.5	1.25	1	1.3	1.3	1.3	1.3	1.3	2.4	2V + 20L
2	*Vigna umbellata*	TRB 160	22	1.2 1.5	1.1 1.25	1.75	1.37	1	1	1	1.3	1.3	1	1	2.2	12V + 10L
3	*Vigna umbellata*	RBS 35	22	1.2	1.6	1	1	1	1	1	1.15	1.3	1.3	1	2.8	12V + 10L
4	*Vigna umbellata*	IC 551699	22	1 1	1.3	1.7	1.15	1.57	1.75	1.1	1	1	1	1	4.0	12V + 10L
5	*Vigna umbellata*	BKSB 192	22	1	1	1	1.25	1.12	1	1	1	1.3	1	1	2.0	16V + 6L
6	*Vigna umbellata*	RBS 53	22	1.2	1.1	1.25	1.25	1	1	1	1	1.3	1.3	1	2.3	12V + 10L
7	*Vigna umbellata*	IC 55440	22	1.57 1.42	1.2	1.35	1.12	1.12	1.12	1	1	1	1.15	1.3	3.0	6V + 16L
8	*Vigna umbellata*	IC 176563	22	1.14 1.28	1.6	1.1	1.25	1.6	1.3	1	1	1.6	1.3	1	2.6	8V + 14L
9	*Vigna umbellata*	EC 97882	22	1.7	1.47	1.2	1.37	1.2	1	1	1.65	1.3	1.3	1	2.3	8V + 14L
10	*Vigna umbellata*	BKSB 194	22	1	1	1	1.3	1.3	1.3	1.3	1.3	1	1	1	3.0	12V + 10L
11	*Vigna aconitifolia*	IC 36157	22	1.33	1.9	2	1.5	1.1 1.5	1.5	1.5	1.5	1	1	1	1.75	6V + 16L
12	*Vigna aconitifolia*	VDV 6175	22	1.5 1.66	2.5	1.33	2	1.5	1.5	1.5	1.5	1.25	1	1	2.5	4V + 16L + 2J
13	*Vigna aconitifolia*	IC 472147	22	2	2.5	2.5 2	1	1	1	1.5	1.75	1.5	1	1	2.25	10V + 12L
14	*Vigna aconitifolia*	RM 040	22	1.5	1.66	3 2	1.5	2	2	1.75	1.25	1.5	1.5	1	2.5	2V + 18L + 2J
15	*Vigna aconitifolia*	IC 39809	22	1.57	2	1.66	1.33	2	1.16	1	1.25	1.25	1.5	1	3.0	4V + 18L
16	*Vigna aconitifolia*	IC 285159	22	1.33	2	1.5	1.5	1	1.5	1.25	1	1	1	1	3.5	10V + 12L
17	*Vigna aconitifolia*	IC 36592	22	1.75 1.6	1.62	1	1	1.66	1	1	1.33	1.33	1	2	2.16	10V + 12L
18	*Vigna aconitifolia*	IC 472173	22	2.4 1.8	1.83 2	1.86	1.25	1.12	1	1	1	1	1	1	4.25	14V + 8L
19	*Vigna aconitifolia*	IC 39713	22	2	2.5	1	2	1	1.5	1.5	1.5	1.5	1.5	1	2.25	6V + 16L
20	*Vigna aconitifolia*	IC 36562	22	1.58	3	1.49	1.33	1	1	1	1.5	1.5	1.5	1	2.5	8V + 14L

**Table 2.** Karyotype formulae and characteristics in the studied taxa of *Vigna*. SC the shortest chromosome length; LC the longest chromosome length; CL mean length of chromosome; CI mean centromeric index; SD standard deviation; CV_CL_ component expressing the relative variation in chromosome length; M_CA_ mean centromeric asymmetry.

Sl. no.	Accessions no.	2n	Range SC-LC (μm)	Ratio LC/SC	CL (μm) Mean (± SD)	CI Mean (± SD)	CV_CL_	M_CA_
1	BKSB 205	22	17-7	2.4	9.13 (± 2.76)	42.87 (± 3.47)	30.25	14.30
2	TRB 160	22	11-5	2.2	7.95(± 1.55)	45.22 (± 4.65)	19.59	9.09
3	RBS 35	22	17-6	2.8	9.18(± 2.95)	46.93 (± 3.85)	32.19	6.18
4	IC 551699	22	24-6	4	11.5(± 4.3)	45.59 (± 5.03)	37.4	9.06
5	BKSB 192	22	12-6	2	8.45(± 1.78)	48.57 (± 2.49)	21.16	2.81
6	RBS 53	22	14-6	2.3	8.40(± 1.74)	47.09 (± 2.91)	20.72	5.78
7	IC 55440	22	18-6	3	9.31(± 2.99)	46.55 (± 2.92)	32.13	6.49
8	IC 176563	22	16-6	2.6	9.18(± 2.66)	44.29 (± 4.65)	29.02	11.37
9	EC 97882	22	14-6	2.3	9.18(± 2.27)	44.94 (± 3.94)	24.82	55.07
10	BKSB 194	22	18-4	3	7.09(± 1.62)	46.75 (± 3.55)	22.86	6.49
11	IC 36157	22	7-4	1.75	5.22 (± 1.04)	42.44 (± 6.07)	19.92	15.32
12	VDV 6175	22	10-4	2.5	5.68 (± 1.54)	40.76 (± 7.06)	27.25	18.55
13	IC 472147	22	9-4	2.25	5.81 (± 1.36)	41.61 (± 8.54)	23.54	16.51
14	RM 040	22	10-4	2.5	6.09 (± 1.67)	38.78 (± 6.45)	27.52	22.88
15	IC 39809	22	12-4	3	6.72 (± 2.02)	42.04 (± 6.17)	30.12	16.22
16	IC 285159	22	7-2	3.5	4.68 (± 1.25)	43.63 (± 5.72)	26.85	10.8
17	IC 36592	22	11-6	2.16	7.95 (± 1.60)	43.89 (± 6.32)	20.22	12.21
18	IC 472173	22	17-4	4.25	8.59 (± 3.66)	44.99 (± 7.29)	42.64	10.14
19	IC 39713	22	9-4	2.25	5.72 (± 1.28)	40.47 (± 6.85)	22.44	19.05
20	IC 36562	22	10-4	2.5	6.22 (± 1.47)	43.34 (± 8.09)	23.68	15.13

Telocentric chromosomes were absent in both the taxa studied. Heteromorphic chromosomes were observed in some of the *Vigna umbellata* accessions: BKSB 205 (1^st^ pair, Fig. 2a), TRB 160 (1^st^ and 2^nd^ pair, Fig. 2b), IC 551699 (1^st^ pair, Fig. 2d), IC55440 (1^st^ pair, Fig. 2g) and IC 176563 (1^st^ pair, Fig. 2h). In *Vigna aconitifolia* heteromorphic chromosomes were found in IC 36157 (5^th^ pair, Fig. 2k), VDV 6175 (1^st^ pair, Fig. 2l), IC 472147 (3^rd^ pair, Fig. 2m), RM040 (3^rd^ pair, Fig. 2n), IC 36592 (1^st^ pair, Fig. 2q) and IC 472173 (1^st^ and 2^nd^ pair, Fig. 2r) accessions. Nucleolar Organizing Regions (NORs), as a secondary constriction/satellites, were observed in *Vigna umbellata* accessions RBS 35 (1^st^ pair, Fig. 2c), IC 551699 (2^nd^ pair, Fig. 2d), and EC 97882 (3^rd^ and 4^th^ pair, Fig. 2i). *Vigna umbellata* was characterized by the presence of both metacentric and submetacentric chromosomes and two *Vigna aconitifolia* accessions (VDV 6175 and RM 040) were characterized by the presence of distinct subtelocentric chromosome, though their position differed in karyotype. The remaining accessions were devoid of any subtelocentric chromosome.

According to the scatter plot obtained by CV_CL_ vs. M_CA_, BKSB 192 (*Vigna umbellata*) and EC 97882 (*Vigna umbellata*) showed the lowest (2.81) and highest (55.07) M_CA_ respectively (Fig. 4). Furthermore IC 285159 (*Vigna aconitifolia*) and RM 040 (*Vigna aconitifolia*) showed lowest (10.8) and highest (22.88) M_CA_ values. In *Vigna umbellata* TRB 160 and IC 551699 exhibited lowest (19.59) and highest (37.4) CV_CL_ values. Among *Vigna aconitifolia* accessions IC 36157 and IC 472173 had shown lowest (19.92) and highest (42.64) CV_CL_ values.

**Figure 3.**
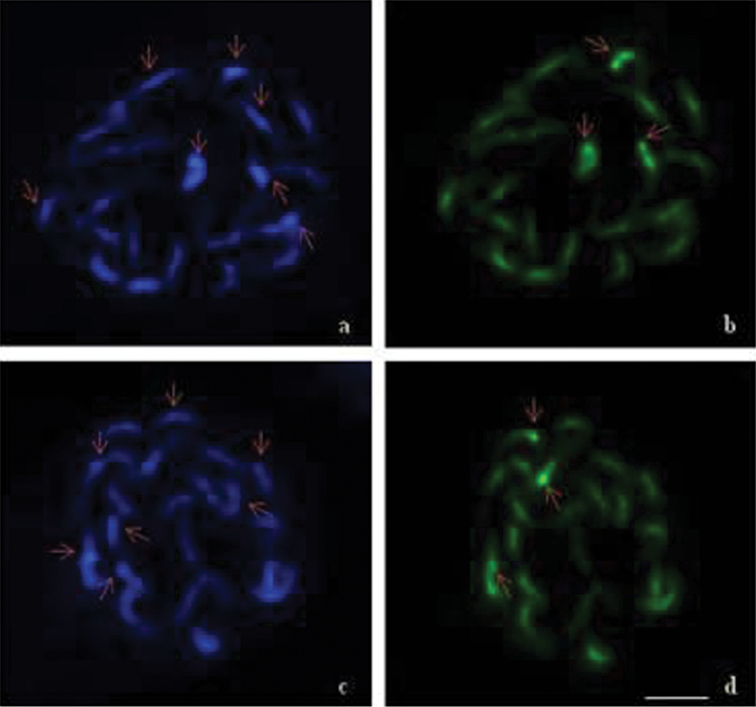
Differentially stained mitotic chromosomes complements **a–b** *Vigna umbellata* **c–d** *Vigna aconitifolia*. Arrows indicate CMA^+^ and DAPI^+^ sites. Scale bar = 5 µm in all the figures.

A comparative account of heterochromatin distribution pattern within the chromosome complements in *Vigna umbellata* and *Vigna aconitifolia* has been summarized in Table 3 and the data have been illustrated in Fig. 3. The CMA_3_^+^ and DAPI^+^ binding sites were found either in terminal or in interstitial regions, in both the taxa studied. *Vigna umbellata* had more of DAPI^+^ sites 3.1(± 1.9) in the interstitial region of the chromosomes and the terminal binding sites were 1.8(± 0.6). The number of chromosomes showing different CMA^+^ and DAPI^+^ sites also ranged from 2–7 in this species. On the other hand, in *Vigna aconitifolia* the heterochromatin block comprised more of CMA^+^ binding sites 2.9(± 1.3), which were found in the terminal region of the chromosomes while 2(± 1.2) binding sites were interstitial in position. The number of chromosomes showing CMA^+^ sites ranged from 3–7, while those showing the DAPI^+^ sites ranged from 3–8.

**Figure 4.**
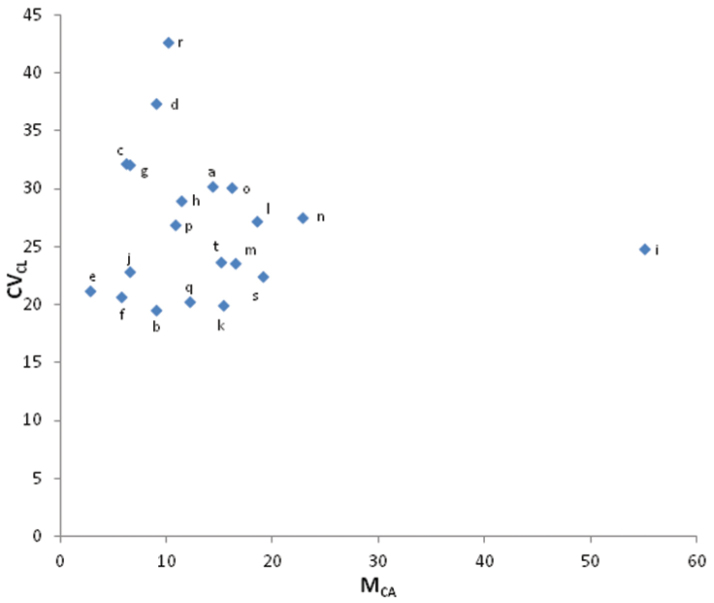
Scatter plot based on the karyotype parameters M_CA_ (x axis) vs. CV_CL_ (y axis) **a** BKSB 192 **b** RBS 53 **c** RBS 35 **d** BKSB 194 **e** IC 55440 **f** IC 551699 **g** TRB 160 **h** IC 472173 **i** IC 285159 **j** IC 176563 **k** IC 36592 **l** BKSB 205 **m** IC 36562 **n** IC 36157 **o** IC 39809 **p** IC 472147 **q** VDV 6175 **r** IC 39713 **s** RM 040 **t** EC 97882.

**Table 3.** Distribution of CMA^+^ and DAPI^+^ sites in the chromosomes of *Vigna* species.

Species	Mean± SD of CMA^+^ sites in chromosomes		Mean± SD of DAPI^+^ sites in chromosomes		Range of CMA^+^ sites Terminal	Range of DAPI^+^ sites Interstitial
	Terminal	Interstitial	Terminal	Interstitial		
*Vigna umbellata*	1.7 ± 0.8	2.1 ± 0.8	1.8 ± 0.6	3.1 ± 1.9	1.7 ± 0.8	2.1 ± 0.8
*Vigna aconitifolia*	2.9 ± 1.3	2 ± 1.2	2.7 ± 0.7	2.3 ± 0.8	2.9 ± 1.3	2 ± 1.2

## Discussion

The present data, combined with the chromosome counts available from the literature confirm the somatic chromosome number of 2*n* = 22 for both species, *Vigna umbellata* and *Vigna aconitifolia*. Such observation received support from reports of Singh and Roy (1970), Rao and Chandel (1991), Rao and Raina (2004), Shamurailatpam et al. (2012). The presence of subtelocentric chromosomes in *Vigna aconitifolia* accessions is in agreement with the earlier report of Sinha and Roy (1979).

All the accessions of *Vigna umbellata* and *Vigna aconitifolia* have shown no deviation in somatic chromosome numbers and overall karyotype appearance. However, *Vigna umbellata* had a higher degree of karyotype asymmetry as compared to *Vigna aconitifolia*, suggesting structural rearrangements in karyotypes. Hence, the observed karyotype variation is likely to have originated by structural changes in chromosomes vs. duplication, deletions, interchanges and inversions (Stebbins 1971, Rao and Chandel 1991). Thus, structural alteration of the chromosomes involving centric fusion and centromere repositioning might have influenced the speciation in genus *Vigna*.

Due to the very small size of chromosomes accompanied by technical difficulties, the nucleolus organisers among the chromosome complements could not be clearly resolved. Other cytogenetic techniques such as silver staining and fluorescence *in situ* hybridization (FISH) can be useful in detecting NOR-loci on chromosomes.

The DAPI^+^ binding sites in chromosomes, which are indicative of AT-rich region, were recorded in the interstitial regions of chromosomes in *Vigna umbellata*. However CMA^+^ sites, found mostly in *Vigna aconitifolia* chromosomes, suggest that the heterochromatin blocks were rich in GC base composition at terminal regions of chromosomes. The higher distribution of AT- and GC- repetitive sequence in heterochromatin blocks is probably reflecting the processes of divergent evolution of repetitive sequences, in heterochromatin regions of *Vigna* species (Shamurailatpam et al. 2014).

In the course of evolution, most of the heterochromatin regions tend to increase (Ikeda 1988), this phenomenon is also observed in *Vigna* (Shamurailatpam et al. 2015). Certain genera such as *Vicia*, *Phaseolus*, *Sesbania*, *Cicer* and *Vigna* (Greilhuber 1975, Zheng et al. 1991, Forni-Martins et al. 1994, Galasso et al. 1996a, b, Forni-Martins and Guerra 1999) showed a heterochromatin-rich chromosome configuration, that might have been involved in diversification of this genus. *Vigna umbellata*, which is domesticated extensively in the sub tropical hilly and moist regions of North-east India, had its heterochromatin blocks rich in AT content with fewer GC base pairs. On the contrary, more GC content in heterochromatin blocks was observed in *Vigna aconitifolia*, which is acclimatized to the arid and semi-arid region of tropical Western plains of India, helping the species to overcome adverse climatic conditions of Indian desert. Our observations in this regard constitute a first attempt to probe the role of heterochromatin distribution pattern, if any, in species differentiation of plant groups.
